# Tailoring the Microstructure of Porous Carbon Spheres as High Rate Performance Anodes for Lithium-Ion Batteries

**DOI:** 10.3390/ma16134828

**Published:** 2023-07-05

**Authors:** Zikun Liang, Ang Li, Kaiming Deng, Bo Ouyang, Erjun Kan

**Affiliations:** Department of Applied Physics, Faculty of Science, Nanjing University of Science and Technology, Nanjing 210094, China

**Keywords:** carbon nanospheres, microstructure regulation, Li^+^ storage behavior, excellent capacity and stability

## Abstract

Benefiting from their high surface areas, excellent conductivity, and environmental-friendliness, porous carbon nanospheres (PCSs) are of particular attraction for the anodes of lithium-ion batteries (LIBs). However, the regulation of carbon nanospheres with controlled pore distribution and graphitization for delivering high Li^+^ storage behavior is still under investigation. Here, we provide a facile approach to obtain PCSs with different microstructures via modulating the carbonization temperatures. With the processing temperature of 850 °C, the optimized PCSs exhibit an increased surface area, electrical conductivity, and enhanced specific capacity (202 mA h g^−1^ at 2 A g^−1^) compared to the PCSs carbonized at lower temperatures. Additionally, PCSs 850 provide excellent cyclability with a capacity retention of 83% for 500 cycles. Such work can pave a new pathway to achieve carbon nanospheres with excellent performances in LIBs.

## 1. Introduction

With the development of human civilization, there is a great interest in applying renewable resources for energy storage technology, typically lithium-ion batteries (LIBs), which pose new requirements and challenges to electrode materials [1,2,3]. Benefiting from wide distribution, little pollution, and abundant sources, carbon-based materials, especially graphite-based frameworks, have received considerable attention in LIBs. To date, based on tremendous investigation, graphite electrodes have been confirmed to deliver great capability and cyclability, along with a much-enhanced LIB performance with graphite-based composite electrodes, such as Si-graphite [4,5] and metal oxide-graphite [6,7]. With superior charge transfer behavior, graphite usually improves the composite conductivity to enhance overall performance. Typically, an Si-graphite composite is capable of delivering a specific capacity of 2426 mA h g^−1^, compared to 831 and 102 mA h g^−1^ for the initial Si and graphite [8]. Although much progress has been made in graphite-based composite electrodes, complicated and environmentally hazardous fabrication procedures restrict the use of these composites in the laboratory. Additionally, the resultant composites usually deliver excessive pollution compared to pure carbon. Hence, it is still necessary to develop carbon materials with satisfactory LIB behavior.

To date, many strategies have been provided to fabricate carbon materials with reduced expense and enhanced capability. Among different approaches, the carbonization of biomass has been regarded as an eco-friendly method since biowastes could serve as the renewable carbon precursor. Additionally, most biomasses provide porous structures, which is advantageous to the Li^+^ interaction towards internal carbon and hence leads to improved LIB performance. Various biowastes have been utilized as carbon precursors such as dead leaves [9], plant wastes [10], coconut oil [11], seaweed [12], etc. Typically, Lu et al. reported N, O-co-doped microporous carbon derived biomass waste with enhanced supercapacitive behavior [13]. Zhang et al. provided glucose-derived hollow microspheres for improved Li^+^ storage [14]. Although such a technique has delivered great progress in fabricating porous carbon electrodes, most work has mainly achieved carbon frameworks with a raised graphite proportion via excessive temperature, typically higher than 1000 °C [15,16]. Moreover, multi-step processes are commonly required to modulate the porosities of carbon frameworks, which inevitably limits the industrial utilization of biomass-derived carbon. Hence, it is important to provide a facile strategy to modulate carbon nanostructures based on reduced temperature.

Here, we report a simple approach to tailor carbon materials. With glucose nanospheres as the precursor, the size of nanosphere can be easily modulated along with simultaneous control of graphitization based on the calcination temperature. By tuning the reaction temperature, the porous structure, surface area, and graphitization can be precisely modulated. As an anode in LIBs, the 850 °C-treated porous carbon sphere (PCS 850) exhibits superior electrochemical performance compared to that of the 450 °C and 650 °C-treated PCSs. Notably, the PCSs 850 achieve a stable capacity of 202 mA h g^−1^ at 2 A g^−1^ for 500 cycles with a capacity retention of 83%. The excellent rate performance should be attributed to the improved porous structure along with enhanced graphitization degree, which enables facile Li^+^ transfer between the electrolyte and electrode. Such work paves an environmentally compatible pathway to controllably achieve carbon nanospheres for LIB applications.

## 2. Experimental Section

### 2.1. Materials Synthesis

The PCSs were synthesized by a two-step carbonization and calcination process. Firstly, carbon nanospheres were fabricated using a simple hydrothermal method as previously reported [17]. Briefly, glucose (5.4 g) was dissolved in ultrapure water (60 mL) to form a clear solution by constantly stirring for 30 min. Then the solution was transferred into a Teflon-lined stainless-steel autoclave (100 mL in capacity). The autoclave was maintained at 180 °C for 4 h, and then naturally cooled to room temperature. The products were separated by centrifugation, followed by washing with deionized water and ethanol for 3 times, respectively. Then the as-prepared carbon nanospheres were dried in an oven at 80 °C for 10 h. Secondly, the as-synthesized carbon nanospheres were calcined in a tube furnace at 450 °C (PCSs 450), 650 °C (PCSs 650), or 850 °C (PCSs 850) for 4 h under an argon atmosphere to produce PCSs with different microstructures.

### 2.2. Characterization

X-ray diffraction (XRD) patterns were obtained using a Bruker D8 diffractometer (Billerica, MA, USA) with a Cu Kα radiation source (λ = 0.15406 nm) operated in the 2θ range from 10° to 65°. Nitrogen (N_2_) adsorption-desorption measurements were performed on a Micrometrics Tristar II instrument (ASAP 3020, Gansu, China) at the testing temperature of 77 K. The surface area and pore size distribution were determined using Brunauer-Emmett-Teller (BET) and Barrett-Joyner-Halenda (BJH) analysis. The BET surface area was calculated using tested data points at a relative pressure of P/P_0_ = 0.05–0.25. The pore size distribution was derived from the adsorption branch with the BJH method. A field emission scanning electron microscope (FESEM, JSM-IT500HR, Tokyo, Japan) and a transmission electron microscope (TEM, Talos F200S, Waltham, MA, USA) were used to obtain the morphologies of the samples. Raman spectra (Aramis, Paris, France) were recorded to identify the graphitization degree of the PCSs. X-ray photoelectron spectroscopy (ThermoFisher, Waltham, MA, USA, ESCALAB Xi+) was employed to measure the surface chemical states and banding energy of the samples. Cyclic voltammetry (CV) was performed at a scan rate of 1.0 mV s^−1^ within the range of 0 to 3 V. Image J (Version 1.53, National Institutes of Health, Bethesda, MD, USA) was taken to calculate the mean diameter of the PCSs, and for each sample, 100 nanospheres were randomly selected for the measurement.

### 2.3. Cell Assembly and Electrochemical Testing

The electrodes were prepared by mixing the PCSs, Super-P carbon black, and a poly (vinylidene fluoride) (PVDF) binder with a mass ratio of 8:1:1. The mixture was dispersed in n-methyl pyrrolidone (NMP) solvent to form a slurry, which was uniformly pasted onto Cu foils with a blade. Then the electrodes were dried under a vacuum at 120 °C overnight. The radius of the electrodes was 7 mm, and the mean areal density was 7.8 mg cm^−2^. CR2032-type coin cells were assembled in a glovebox with metal Li foil as the counter electrode and celgard-2400 as the separator. The electrolyte solution was 1 M LiPF_6_ in a 1:1:1 mixture of ethylene carbonate (EC), dimethyl carbonate (DMC), and diethyl carbonate (DEC). The galvanostatic charge-discharge of the assembled cells was measured at various current densities in the voltage range of 0–3 V vs. Li/Li^+^ by a battery test system (Land CT2001A, Wuhan, China). Electrochemical impedance spectroscopy (EIS) tests were performed on a electrochemical workstation (VSP 300, Paris, France) with an AC signal amplitude of 5 mV in the frequency range of 100 kHz to 100 mHz.

## 3. Results and Discussion

**Figure 1:**
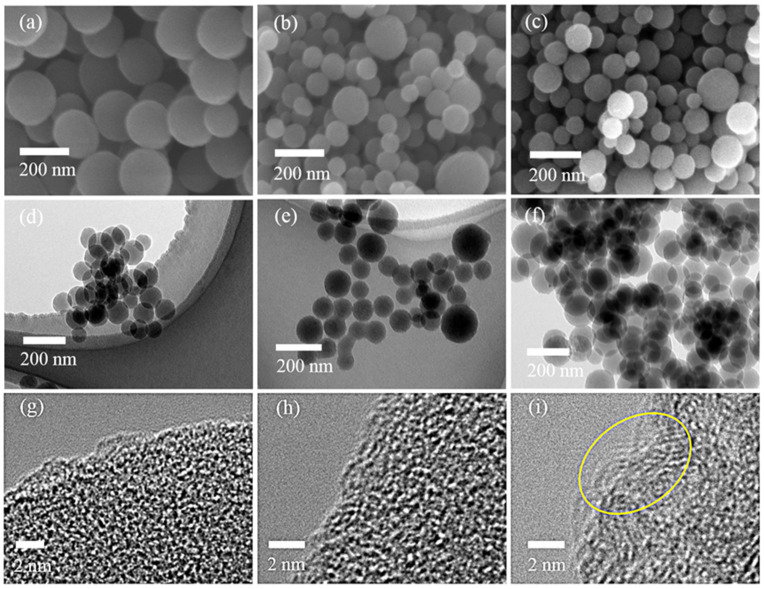
SEM (**a**–**c**), TEM (**d**–**f**), and HRTEM (**g**–**i**) images of PCSs 450, PCSs 650, and PCSs 850, respectively. The yellow cycle in image (**i**) represents the graphitic carbon in PCSs 850.

The SEM and TEM images (Figure 1a–f) show that the as-synthesized PCSs are regular nanospheres. Accordingly, the mean diameter of the PCSs reduces from 183 nm (PCSs 450) to 89 nm (PCSs 650) and 87 nm (PCSs 850) with the increased calcination temperatures (Figure S1). The decreased mean size should be attributed to the gas released from the glucose nanospheres during carbonization, which simultaneously leads to the formation of porous structures [18]. Such phenomenon is also correlated with the reduced yield of PCSs with raised treatment temperatures owing to the increased gaseous release. In terms of HRTEM (Figure 1g–i), the graphitic microregions can hardly be observed in PCSs 450 and PCSs 650, but are apparently found in PCSs 850, which is ascribed to the enhanced carbonization temperature. The graphitic carbon embedded in amorphous carbon can facilitate the transfer of electrons, which is conducive to the improvement of rate performance.

**Figure 2:**
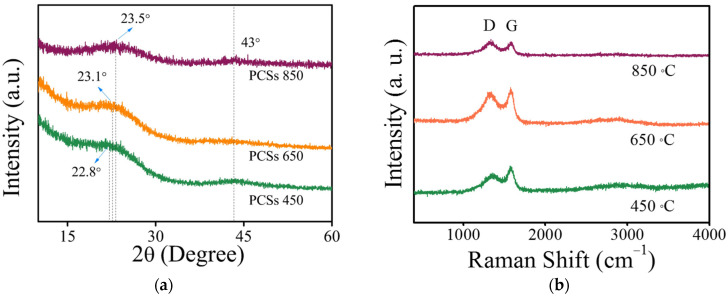
(**a**) XRD patterns and (**b**) Raman spectra of different carbon spheres.

An XRD pattern was performed to detect the crystal structure of the different PCSs. All samples provided two well-defined peaks at about 23° and 43° (Figure 2a), which correspond to the (002) and (100) planes of graphite, respectively. Noticeably, the broadened peaks at 23° indicate that the PCSs mainly consist of both amorphous and graphitized carbon. The increase in graphitization degree from PCSs 450 to PCSs 650 and PCSs 850 leads to the shift of the (002) band from 22.8° to 23.1° and 23.5°, which corresponds to 0.389, 0.384, and 0.378 nm of graphite (002), respectively. By comparing these values with ideal graphite (0.335 nm), it is confirmed that the graphitization degree gradually increases with the raised carbonization temperature [19]. Raman spectra were also employed to detect the structural changes in the PCSs (Figure 2b). Two characteristic peaks around 1338 and 1590 cm^−1^ could be ascribed to a D band arising from the defects and disorders in the carbonaceous materials and a G band from the stretching mode of C=C bonds of typical graphite, respectively [20]. The intensity ratios (I_D_/I_G_) of PCSs 450, PCSs 650, and PCSs 850 increase from 0.87 to 0.95, then to 1.08, indicating the generation of increased surface defects and disordered carbon induced by nanopore formation based on the carbonization conditions from 450 to 850 °C [21]. The enhanced concentration of defects and disorder could not only facilitate Li^+^ transfer but also provide active sites for lithium storage.

**Figure 3:**
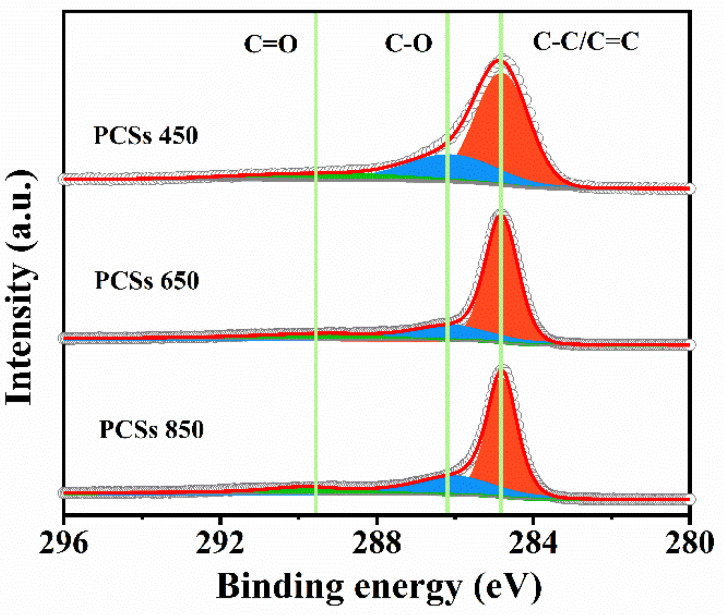
High resolution XPS spectra of C 1s for PCSs.

XPS was conducted to analyze the chemical composition and states on the surface of the PCSs. As shown in Figure S2, the full-scale XPS spectra analysis exhibits that different PCSs primarily consist of carbon and oxygen. The carbon contents of PCSs 450, PCSs 650, and PCS 850 were found to be 84.4%, 90.5%, and 95.7%, respectively, indicating an increase of carbon proportion with the rise of the calcination temperature. On the other hand, the oxygen contents show a declining trend from PCSs 450 to PCSs 850, which is ascribed to the removal of oxygen containing species at the raised carbonization temperatures. The high-resolution XPS spectra of C 1s for different PCSs (Figure 3) display a predominant narrow peak at 284.7 eV, suggesting that the main components on the surface are carbon (C-C/C=C). The peaks at around 286.1 and 289.5 eV reveal the presence of C-O and C=O, respectively [22].

**Figure 4:**
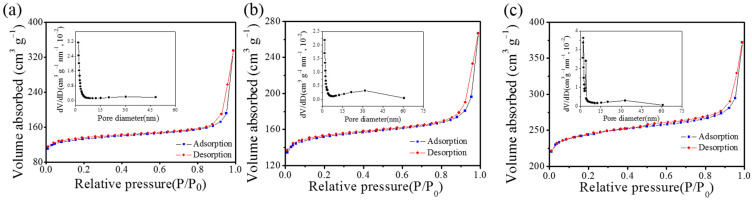
(**a**–**c**) Nitrogen adsorption-desorption isotherms and Barret-Joyner-Halenda (BJH) pore size distribution curves of PCSs 450, PCSs 650, and PCSs 850, respectively.

To further investigate the structural details, N_2_ adsorption-desorption isotherms were collected. As shown in Figure 4, all the samples are type-IV curves. Based on the BET results, the PCSs 850 provides the highest specific surface area of 729 m^2^ g^−1^, while PCSs 450 and PCSs 650 show reduced specific surface areas of 406 and 466 m^2^ g^−1^, respectively. The high specific area of PCSs 850 indicates improved contact with the electrolyte when used as anodes for LIBs. By using the BJH method, the pore size of the PCSs was calculated accordingly. It can be found that all samples contain mesopores (~15 nm) and micropores (~2 nm). Such meso-micro hierarchical porous structures promote charge transfer kinetics and provide additional storage sites [23]. Since the number of micropores is commonly proportionally related to the total specific surface area, it induces increased micropores formed in PCSs 850 during thermal treatment. Therefore, by applying different calcination temperatures, the microstructure of PCSs in terms of specific surface area and pore distribution can be successfully tuned. Detailed information of the BET and BJH results is summarized in Table 1.

**Figure 5:**
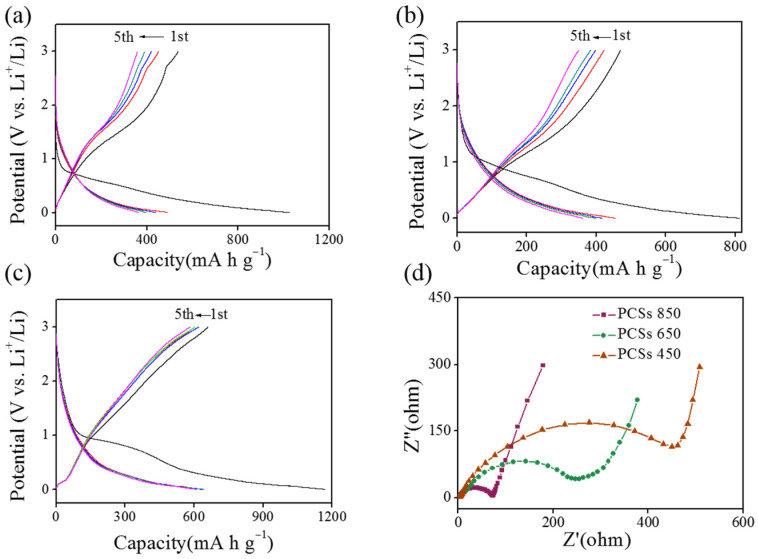
(**a**–**c**) Charge/discharge voltage profiles between 0 and 3 V at a current density of 0.1 A g^−1^ for PCSs 450, PCSs 650, and PCSs 850, respectively. (**d**) EIS spectra of the as-synthesized PCSs.

Figure 5a–c illustrate the 1st to 5th charge-discharge profiles of PCSs 450, PCSs 650, and PCSs 850 at the current density of 0.1 A g^−1^, respectively. It can be observed that all samples exhibited irreversible capacity loss during the initial cycle, which is attributed to the formation of the solid electrolyte interphase (SEI) [24]. From the second cycle, the capacities of the PCSs significantly decrease, indicating the formation of an SEI layer on the PCS surfaces during the first cycle. Furthermore, all the PCSs display voltage profiles with a slope between 0–1 V during the charge-discharge process, unlike commercial graphite which has a low lithiation potential at ~0.3 V. This enhanced lithiation potential makes PCSs the safe anodes for lithium-ion batteries. EIS is a useful method to investigate the electrode process. The Nyquist plots of PCSs 450, PCSs 650, and PCSs 850 are presented in Figure 5d. All samples display a depressed semicircle in the high-frequency region and a straight line in the low-frequency region. The semicircle represents the charge-transfer resistance (R_ct_) between the electrolyte and the electrode, and the sloping line is assigned to the Warburg resistance (Z_w_), which is related to diffusion. It is evident that PCSs 850 manifest the smallest R_ct_ compared with the other electrodes, indicating the best electron transfer capability [25]. The improved conductivity should be ascribed to the raised content of graphic carbon in PCSs 850. Meanwhile, PCSs 850 show the smallest Z_w_, which is ascribed to the large amount of micropores which facilitate Li^+^ transfer. The equivalent circuit model and the fitted impedance parameters of the PCSs are presented in Figure S3 and Table S1.

**Figure 6:**
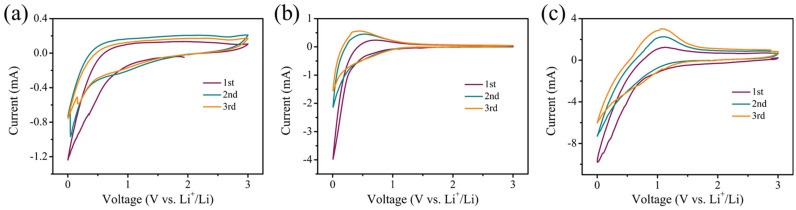
(**a**–**c**) CV profiles of the PCSs 450, PCSs 650, and PCSs 850, respectively.

CV was performed to gain insight into the differences in charge storage behavior of the PCSs. As depicted in Figure 6, all the electrodes exhibit a distinct cathodic peak at 0.05 V, which is ascribed to the characteristic SEI formation of porous carbon [26]. With the apparent intensity difference of cathodic peaks between the first and the subsequent cycles, it is confirmed that a large irreversible capacity loss occured during the lithiation process. The irreversible capacity loss should be attributed to the side reactions and the formation of SEI, which is consistent with the results obtained from the galvanostatic charge-discharge tests. In comparison, the second and third cycles in the CV curves of the different PCS electrodes gradually overlay, indicating reduced irreversible capacity loss during subsequent cycles. During the charging process, all PCSs electrodes deliver a broad band between 0.4 and 1.1 V, which is attributed to the lithium extraction from the PCS electrodes.

**Figure 7:**
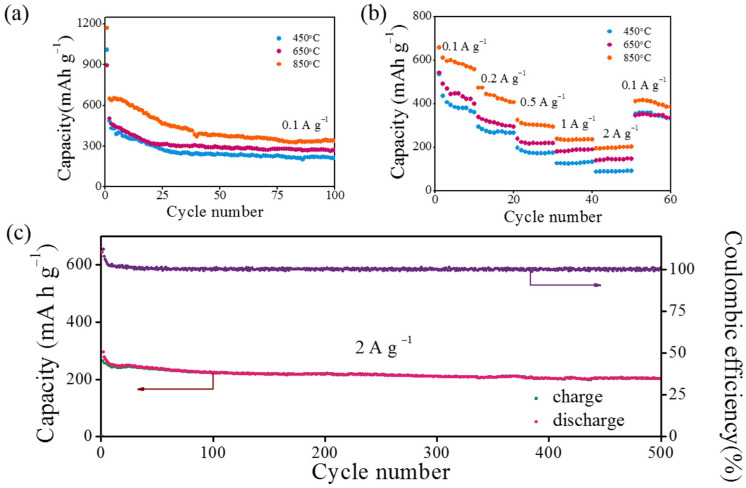
(**a**) Cycling performance of different-temperature-treated carbon spheres at a constant current density of 0.1 A g^−1^ for 100 cycles. (**b**) Multirate performance of the spheres at different current densities from 0.1 A g^−1^ to 2 A g^−1^. (**c**) Long-term cycling performance of 850 °C treated carbon spheres at a high current of 2 A g^−1^.

Figure 7a shows the cycling performance of the PCSs at the current of 0.1 A g^−1^ for 100 cycles. The specific capacities of all the samples show a decrease in the first 30 cycles and then tend to stabilize, which is characteristic of porous carbon materials [27,28,29]. After 100 cycles, the specific capacities of PCSs 450, PCSs 650, and PCSs 850 are 215, 268 and 339 mA h g^−1^, respectively. The enhanced capability of PCSs 850 can be ascribed to the increased surface area along with the enhanced graphitization degree, similar with prior work [30,31]. Considering the high rate capabilities needed for high power applications, we investigated the rate performances of the PCSs 450, PCSs 650, and PCSs 850 at different currents, as shown in Figure 7b. With the current densities increasing from 0.1 to 2 A g^−1^, the specific capacities gradually decrease accordingly [25]. As the test current is regularly returned to a low current rate of 0.1 A g^−1^, the specific capacities are restored to values close to those of previous measurements, indicating the good stability of PCSs. Noticeably, PCSs 850 provide a capacity preservation of 33% from 0.1 to 2 A g^−1^, higher than the 25% of PCSs 450, which indicates reduced amorphous carbon proportion and thus enhanced charge transfer behavior. Indeed, such a capacity fading in PCSs 850 is still not comparable to a pure graphitic structure since there is much amorphous carbon, but it is comparable to other carbon nanostructures in prior work [32,33]. Overall, the PCSs 850 show the best rate performance, which should be attributed to the enhanced specific area that shortens the charge transfer length, the increased micropores that provide raised storage sites, and the great conductivity that facilitates the transport of electrons in the solid. Furthermore, the long-term cycling performance of PCSs 850 was assessed at the high current of 2 A g^−1^. As shown in Figure 7c, PCSs 850 obtained a high capacity of 202 mA h g^−1^ after 500 cycles, and its Coulombic efficiency kept nearly 100% after the first several cycles, implying its great potential as high rate performance anodes for lithium-ion batteries.

## 4. Conclusions

In summary, we have proposed a facile carbonization strategy to control carbon nanospheres with controllable graphitization degree and porous structures. With increased processing temperatures, PCSs deliver increased surface area along with increased micropores, leading to an enhanced Li^+^ storage behavior. Typically, the PCSs 850 achieve a stable capacity of 202 mA h g^−1^ at 2 A g^−1^ for 500 cycles with a capacity retention of 83%. Such great performance should be attributed to the optimized porous structure and enhanced graphitization degree, which enables easy transport of Li^+^ between electrode and electrolyte. This work paves an environmentally-compatible pathway to controllably achieve porous carbon nanospheres for LIB applications.

## Figures and Tables

**Table 1:** Summary of parameters of the as-prepared PCSs according to nitrogen adsorption-desorption isotherms.

Sample	Yield (%)	S_BET_ (m^2^/g)	S_micro_ (m^2^/g)	S_micro_/S_BET_ (%)
PCSs 450	61.1	407	291	71.5
PCSs 650	49.5	466	381	81.8
PCSs 850	42.0	730	604	82.7

## Data Availability

The data presented in this study are available on request from the corresponding author.

## Supplementary Materials

The following supporting information can be downloaded at: https://www.mdpi.com/article/10.3390/ma16134828/s1, Figure S1: Full-scale XPS spectra of PCSs 450, PCSs 650, PCSs 850, respectively; Figure S2: Equivalent circuit models of the PCSs. Figure S3: Equivalent circuit models of PCSs; Table S1: The fitted impedance parameters of the equivalent circuits in Figure 5d.
