# Supplementary material for: Tailoring the Microstructure of Porous Carbon Spheres as High Rate Performance Anodes for Lithium-Ion Batteries
Source: Materials (Basel). 2023 Jul 5;16(13):4828. doi: 10.3390/ma16134828 (PMC10343343; doi:10.3390/ma16134828)
Supplement: Supplementary file 1 [file materials-16-04828-s001.zip › materials-2396805-supplementary.pdf]

Supporting information

Tailoring the Microstructure of Porous Carbon Spheres as High Rate Performance Anodes for Lithium-Ion Batteries

Zikun Liang, Ang Li, Kaiming Deng, Bo Ouyang * and Erjun Kan

Department of Applied Physics, Faculty of Science, Nanjing University of Science and Technology, Nanjing 210094, China

* Corresponding authors

Email address: Bo Ouyang (ouyangboyi@njust.edu.cn)

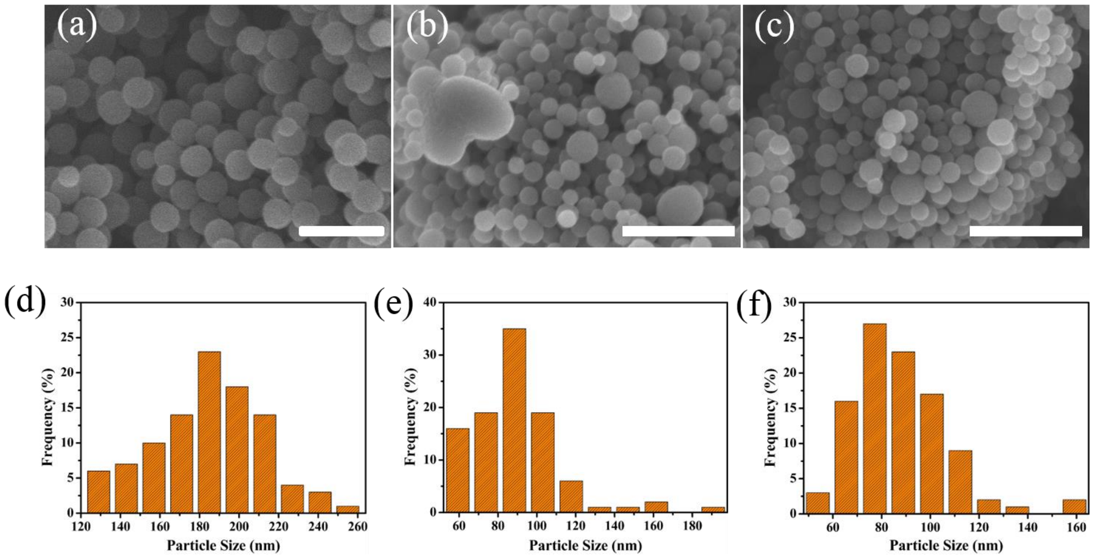

Figure S1. SEM (a-c) and particle size distribution (d-f) of the PCSs 450, PCSs 650, PCSs 850, respectively. The scale bar of the SEM images is 500 nm.

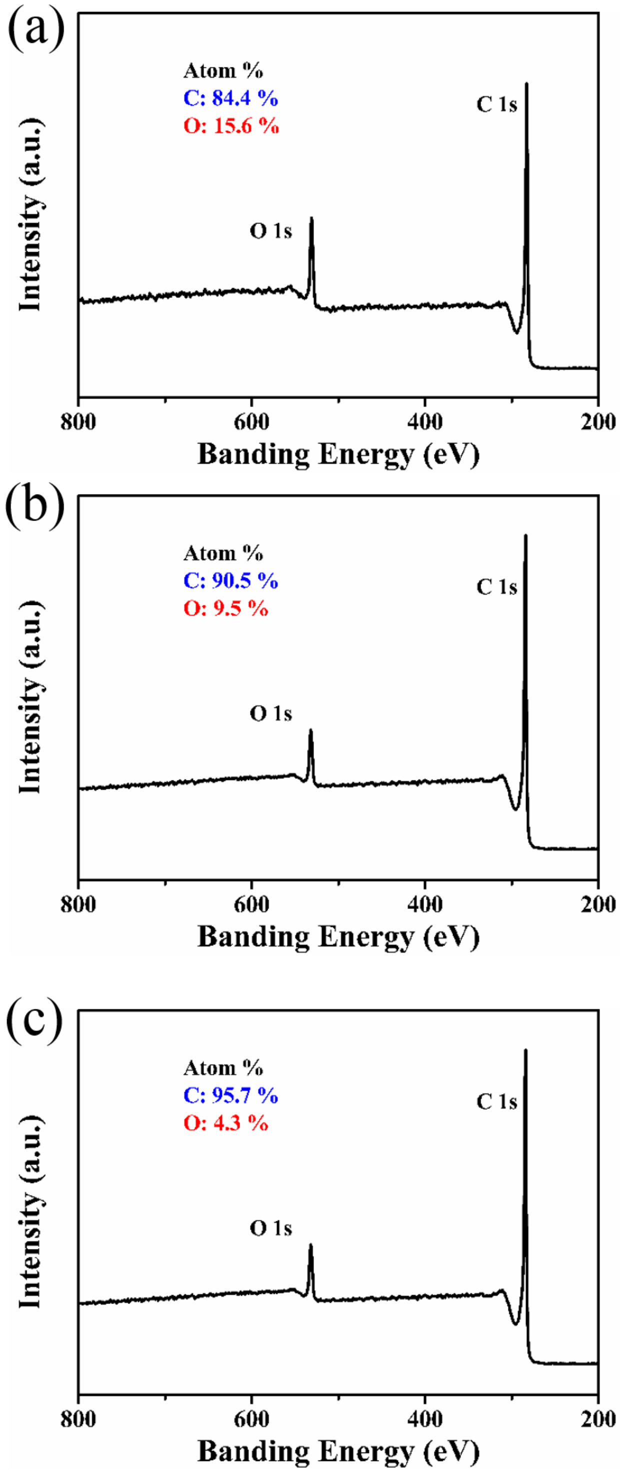

Figure S2. (a-c) Full-scale XPS spectra of the PCSs 450, PCSs 650, PCSs 850, respectively.

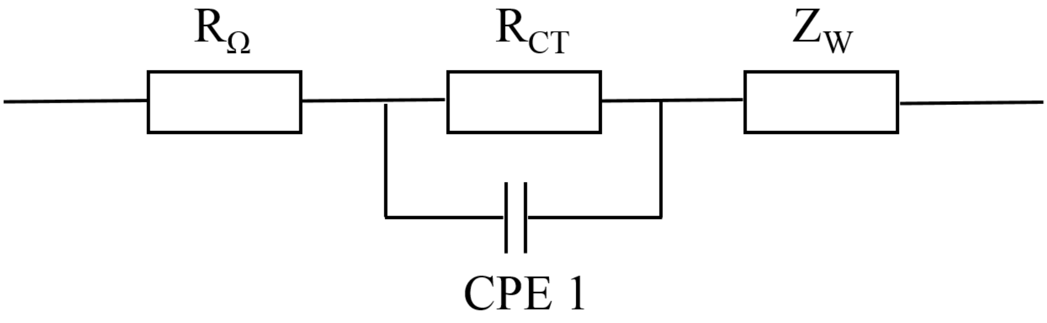

Figure S3. Equivalent circuit models of PCSs.

Table S1. The fitted impedance parameters of the equivalent circuits in Figure 5d.

Samples	R_{Ω} (Ω)	R_{CT} (Ω)	CPE 1 (mF)	Z_W (Ω)
PCSs 450	6.25	63.28	0. 36	0.0585
PCSs 650	6.22	45.36	0. 27	0.0429
PCSs 850	6.17	28.72	0. 32	0.0172
